# A Differentiable, GPU-Accelerated Finite-Element Formulation in JAX for Large-Deformation Shells with Contact: Application to Patient-Specific Aortic Valve Simulation

**Published:** 2026-10-27

**Authors:** Mohsen Nakhaei, Alison M. Pouch, Matthew A. Jolley, Wensi Wu

**Affiliations:** a Department of Pediatrics, Division of Cardiology, Children’s Hospital of Philadelphia, Philadelphia, PA, USA; b Department of Radiology and Bioengineering, University of Pennsylvania, Philadelphia, PA, USA; c Department of Anesthesiology and Critical Care Medicine, Children’s Hospital of Philadelphia, Philadelphia, PA, USA; d Department of Mechanical Engineering and Applied Mechanics, University of Pennsylvania, Philadelphia, PA, USA; e Cardiovascular Institute, Children’s Hospital of Philadelphia, Philadelphia, PA, USA

**Keywords:** Differentiable finite elements, Automatic differentiation, Matrix-free Newton–Krylov, Finite-strain shells, Contact mechanics, Patient-specific aortic valve

## Abstract

Patient-specific aortic-valve models can help assess how tissue properties and geometry influence leaflet deformation and coaptation, with potential to inform intervention planning. Large deformations, near-incompressibility, and evolving contact make these simulations computationally demanding and can challenge nonlinear convergence. Differentiable, GPU-accelerated methods offer efficient derivative evaluation and parallel computation, but their matrix-free application to soft-tissue shells with contact remains less established. We developed a forward solver using tatva’s energy-based finite-element framework in JAX. The solver combines finite-strain extensible-director shells, hyperelastic constitutive models, and a finite-range repulsive contact energy. Nonlinear equilibrium is solved using a regularized, matrix-free Newton–Krylov method with two-level preconditioning. Within this solver, automatic differentiation (AD) evaluates tangent–vector products without forming or storing the primary tangent matrix, while GPU execution parallelizes element and quadrature-point computations. Constitutive, shell, contact, and self-contact responses were assessed against analytical, semi-analytical, and published benchmarks. At a contact-free plate equilibrium, implicit sensitivities of the converged apex displacement with respect to constitutive parameters, thickness, and pressure agreed with finite differences from independent nonlinear simulations. Simulations of three patient-specific aortic-valve models (two tricuspid and one bicuspid) achieved final relative equilibrium residuals below 10^−4^. Median GPU runtimes were approximately 15–25 minutes, with 5.3–6.5-fold speedups relative to corresponding CPU runs on the tested hardware. These results provide component-level verification and demonstrate GPU-accelerated forward valve simulation in the tested cases, supporting future gradient-based inverse studies.

## 1. Introduction

Aortic valve (AV) disease is among the most common forms of valvular heart disease and a frequent reason for surgical or transcatheter intervention [1, 2]. Patient-specific computational models offer a means of examining how leaflet structure and deformation influence valve function. These models provide estimates of leaflet stresses and strains that can inform biomechanical assessment of disease and potential interventions [3, 4]. Their clinical value depends on simulation accuracy, computational efficiency, and validation against independent data [5, 6]. During valve closure, thin, nearly incompressible leaflets undergo large deformations that alter their geometry and tangent stiffness, and contact forces develop as the leaflets approach one another [7, 8]. Solving the resulting nonlinear equilibrium equations requires repeated evaluation of forces and their derivatives. A differentiable finite-element formulation supports these calculations in the forward solve and provides a foundation for future gradient-based inverse studies. GPU execution can accelerate element and quadrature-point computations through parallel processing, potentially making repeated patient-specific simulations more practical for clinical assessment and intervention planning.

Automatic differentiation (AD) provides these derivative calculations by applying the chain rule to implemented energy and residual functions [9, 10]. Together with GPU computing, it supports both the training of neural-network finite-element (NNFE) models and Newton-type solution of finite-element equilibrium equations. For example, Meyer et al. [11] developed an NNFE model of three-dimensional solid valve leaflets with multi-body contact. After energy-based training, the network predicts displacements over the pressure range considered. In Newton-type finite-element solvers, AD differentiates the residual with respect to the finite-element unknowns to evaluate tangent operators or tangent–vector products, reducing the need for manually derived expressions [9]. JAX-FEM and torch-fem implement differentiable finite-element formulations for structural analysis and gradient-based optimization [12, 13]. TensorMesh uses tensorized finite-element assembly and sparse linear solvers for GPU-based computation. The associated TensorGalerkin framework supports AD through assembly and adjoint differentiation of sparse linear solves [14]. In biomechanics, PyTorch-FEA applies AD to forward inflation and inverse identification of patient-specific material parameters in the human aorta [15].

Within the JAX ecosystem, tatva uses an energy-centric finite-element formulation in which AD computes residuals and tangent operators from the global energy [10]. This structure allows us to express shell deformation and contact as separate energy terms and combine them before differentiation. When the material or contact energy expressions are modified, AD computes the corresponding residual and tangent contributions without manually deriving new expressions. Tatva provides reusable operators for field interpolation, gradient evaluation at quadrature points, and numerical integration, along with utilities for imposing prescribed boundary values. For equilibrium solves, tatva evaluates tangent–vector products without forming the full tangent matrix and provides graph-coloring-based sparse reconstruction when an explicit sparse tangent is needed. JAX supplies the underlying automatic differentiation, vectorization, and execution on CPUs and GPUs [16]. Together, tatva’s finite-element operators and JAX’s computational capabilities provide a suitable foundation for our valve application.

However, applying these computational capabilities to valve mechanics also requires suitable shell discretization, treatment of evolving contact, and an effective nonlinear solution procedure. In shell discretizations, constraints on shear and through-thickness deformation can introduce artificial stiffness [17, 18]. Large stiffness differences can make the linearized equilibrium equations difficult to solve, making effective preconditioning important for iterative methods [19]. Evolving contact also presents challenges for differentiability because the interacting surface locations and contact status depend on deformation. Closest-point projection requires careful treatment of surface geometry and transitions between face, edge, and vertex projections [20, 21]. Changes in active contact constraints or the selected closest facet can introduce nonsmooth behavior, motivating specialized or regularized contact formulations for sensitivity analysis [22, 23]. Addressing these requirements would support repeated forward simulations and provide a foundation for future gradient-based inverse studies using implicit differentiation [24].

In the present study, we develop shell and contact implementations for large-deformation soft-tissue mechanics within tatva’s energy-based finite-element framework in JAX. Our implementation combines finite-strain extensible-director shells, hyperelastic constitutive models, and a finite-range quartic repulsive contact energy. The three-node shell incorporates mixed interpolation of tensorial components (MITC3) to reduce transverse-shear locking [17] and an enhanced-assumed-strain (EAS) mode to enrich the transverse-normal strain field [18]. Nonlinear equilibrium is solved using a regularized, matrix-free Newton–Krylov method with two-level preconditioning. We first evaluate constitutive response, shell deformation, and contact behavior against analytical and semi-analytical reference solutions and published benchmarks, including a self-contact ring test. We subsequently verify implicit derivatives of the converged apex displacement using a contact-free plate. Finally, we apply the formulation to three patient-specific aortic valves reconstructed from image segmentations and examine mesh and numerical sensitivity. CPU and GPU performance are compared for the plate and valve simulations.

Section 2 presents the mechanical formulations and nonlinear solution strategy. Section 3 presents component verification, CPU–GPU performance comparisons, and patient-specific valve-closure results. Section 4 discusses the main findings and limitations.

## 2. Methods

### 2.1. Governing equations and constitutive models

We model deformable bodies as hyperelastic continua undergoing finite deformation. Let $\Omega_{0}\subset R^{3}$ denote the reference configuration, $X\in \Omega_{0}$ the material coordinates, and φ(X)=X+u(X) the deformation map, where u is the displacement field. The deformation gradient, its Jacobian, and the right Cauchy–Green tensor are

$$F=I+\nabla_{0}u,\;J=det\;F>0,\;C=F^{⊤}F,$$

where $\nabla_{0}$ denotes the gradient with respect to the reference coordinates. The first invariant of C is $I_{1}=tr\;C=\lambda_{1}^{2}+\lambda_{2}^{2}+\lambda_{3}^{2}$. It is equal to the sum of the squared principal stretches and provides the isotropic deformation measure used in the strain-energy functions below.

For fixed external forces, equilibrium requires the first variation of the total potential energy to vanish, consistent with the principle of virtual work. Writing the internal, contact, and external contributions separately,

(1) $$\begin{array}{l} \Pi (u)=\Pi_{int}(u)+\Pi_{c}(u)-\Pi_{ext}(u),\\ \;\Pi_{int}=\Pi_{mat}+\Pi_{d},\;\Pi_{mat}=\int_{\Omega_{0}}\psi (F)\;dV, \end{array}$$

the equilibrium configuration satisfies δΠ=0 for all virtual displacements that vanish where displacements are prescribed. Here, $\Pi_{\text{mat}}$ is the stored strain energy of the material, and $\Pi_{d}$ is the shell drilling-stabilization energy defined in Section 2.2. This numerical penalty stabilizes rotation about the shell’s local normal, which otherwise introduces a zero-energy mode [25]. Together, these terms form the internal energy $\Pi_{\text{int}}$. The term $\Pi_{c}$ is the piecewise-smooth contact energy described in Section 2.3. The term $\Pi_{\text{ext}}$ represents the work of the applied loads. Pressure acts normal to the deformed surface and changes direction as the surface deforms. In the pressure-loaded shell simulations, the loaded-face geometry and pressure force vector are held fixed within each load step and updated between load steps and during final equilibrium refinement, as described in Section 2.4. Equation (1) therefore defines the potential used for fixed-force evaluations; a globally defined potential for the geometry-dependent pressure load is not assumed.

After finite-element discretization, the potential depends on the primary nodal variables U. For shells, these include displacements and rotations, and the potential also depends on the nodal internal variables w that control the thickness stretch and the element-local enhanced-strain variables α. These shell variables are defined in Section 2.2:

(2) $${\tilde{\Pi }}_{h}(U,w,\alpha )=\sum_{e}\sum_{q}\psi_{h}\left(U,w,\alpha ;\xi_{q}\right)\;\omega_{q}\;det\;J_{e}\left(\xi_{q}\right)+\Pi_{d}+\Pi_{c}(U)-\Pi_{ext}(U),$$

where $\psi_{h}$ is the strain-energy density, $\omega_{q}$ and $\xi_{q}$ are the quadrature weights and locations, and $J_{e}$ is the Jacobian of the mapping from element coordinates to the reference configuration. The notation $\omega_{q}$ is used for the quadrature weights to distinguish them from the nodal thickness-stretch variables w.

During each derivative evaluation, w,α, and the pressure force vector are held fixed. For each contact sampling point, automatic differentiation uses the distance to the target facet that is closest at the current configuration. The closest facet may change between evaluations, making the contact contribution piecewise smooth. The equilibrium residual r and tangent–vector product Kv for an arbitrary direction v are,

$$r=\frac{\partial {\tilde{\Pi }}_{h}}{\partial U},\;Kv=\frac{\partial^{2}{\tilde{\Pi }}_{h}}{\partial U^{2}}v.$$

Both quantities are evaluated by automatic differentiation, without a manually derived tangent or assembly of the global tangent matrix K. The shell discretization is described in Section 2.2, and the differentiation and solution procedures are presented in Section 2.4. Because the contact energy is included in the same scalar functional, its contributions to r and Kv are obtained through the same automatic-differentiation procedure, without separate contact-element assembly. During the final equilibrium refinement, the coupling between U and the internal shell variables w and α is also accounted for, as described in Section 2.4. The implementation builds on tatva’s energy-based finite-element approach and its Element base class, using JAX to evaluate the problem-specific energy gradients and tangent–vector products [10].

#### Constitutive models:

Three constitutive models are implemented: a compressible neo-Hookean law for isotropic elasticity, an isotropic Lee–Sacks law for nonlinear strain stiffening, and a neo-Hookean ground matrix with Holzapfel–Gasser–Ogden (HGO) fiber reinforcement for fiber-induced anisotropy. Each model penalizes volume changes through a term proportional to ${\left(ln\;J\right)}^{2}$. Near-incompressibility is therefore imposed approximately through a penalty rather than exactly as a kinematic constraint.

The compressible neo-Hookean strain-energy density is,

(3) $$\psi_{NH}=\frac{1}{2}\mu \left(I_{1}-3-2\;ln\;J\right)+\frac{1}{2}\lambda (ln\;J{)}^{2},$$

where μ and λ are the Lamé parameters. The corresponding infinitesimal bulk modulus is $K_{\text{bulk}}=\lambda +2\mu /3$.

The isotropic Lee–Sacks law [26, 27] augments a neo-Hookean ground matrix with an exponential term that phenomenologically represents the progressive strain stiffening associated with collagen recruitment:

(4) $$\psi_{LS}=\frac{1}{2}c_{0}\left(I_{1}-3-2\;ln\;J\right)+\frac{1}{2}c_{1}\left[exp\left(c_{2}{\left(I_{1}-3\right)}^{2}\right)-1\right]+\frac{1}{2}K(ln\;J{)}^{2}.$$

For the patient-specific valve simulations, the material parameters are $\left(c_{0},c_{1},K\right)=\left(67,\;13,\;5000\right)\;kPa$, with dimensionless $c_{2}=38$. These values were adopted from prior heart-valve studies [27, 28].

To represent anisotropy, each collagen fiber family i, with reference orientation $a_{0i}$, contributes an exponential HGO reinforcement term [29]:

(5) $$\psi_{\text{aniso}}=\sum_{i}\frac{k_{1}}{2k_{2}}\left[exp\left(k_{2}{\left\langle I_{4i}-1\right\rangle }_{+}^{2}\right)-1\right],\;I_{4i}=a_{0i}\cdot Ca_{0i},$$

where $\left\langle x\rangle_{+}=max(x,0\right)$. Here, $a_{0i}$ is a unit vector, $k_{1}$ is a fiber stiffness parameter with units of stress, and $k_{2}$ is a dimensionless stiffening parameter. The complete HGO strain-energy density is,

(6) $$\psi_{HGO}=\psi_{NH}+\psi_{\text{aniso}}.$$

The Macaulay bracket activates each fiber family only when $I_{4i}>1$, corresponding to extension beyond its reference length. Thus, the fibers carry tensile load but do not resist compression. The resulting anisotropic energy is continuously differentiable but not twice continuously differentiable at $I_{4i}=1$. The full fiber invariant $I_{4i}$ is used rather than its isochoric projection [30].

### 2.2. Shell formulation

#### Element and kinematics:

We use three-node triangular shell elements [17]. The element adopts seven-parameter extensible-director shell kinematics [18, 31]. The current position of a point in the shell is described by

(7) $$x(\xi ,\zeta )=x_{m}(\xi )+\frac{t}{2}\zeta \tilde{d}(\xi ),\;\zeta \in [-1,\;1],$$

where ξ denotes the in-plane element coordinates, $x_{m}$ is the deformed midsurface, t is the reference thickness, and ζ is the normalized through-thickness coordinate. The scaled director field is interpolated from the three element nodes as

(8) $$\tilde{d}(\xi )=\sum_{I=1}^{3}N_{I}(\xi )\lambda_{t,I}d_{I},$$

where $N_{I}$ is the linear shape function, $d_{I}$ is the unit director, and $\lambda_{t,I}$ is the thickness stretch at node I. Thus, the current nodal thickness is $t\lambda_{t,I}$.

Each node carries seven kinematic parameters: three translational degrees of freedom $u_{I}$, three rotational parameters $\theta_{I}$ that define the director orientation, and one scalar parameter $w_{I}$ that controls the thickness stretch. The translation and rotation parameters are primary nodal unknowns, whereas $w_{I}$ is treated as an internal nodal variable. Finite director rotations are represented using the exponential map [32],

(9) $$d_{I}=exp\left({\left[\theta_{I}\right]}_{\times }\right)\;D_{I},$$

where $D_{I}$ is the reference unit director and ${\left[\theta_{I}\right]}_{\times }$ is the skew-symmetric matrix associated with $\theta_{I}$. This rotation preserves the unit length of $d_{I}$. The nodal thickness stretch is defined as

(10) $$\lambda_{t,I}=exp\left(w_{I}\right),$$

where $w_{I}$ is the nodal logarithmic thickness-stretch parameter. Shared nodal director and thickness-stretch values make the interpolated scaled director field continuous across element boundaries. The exponential definition guarantees $\lambda_{t,I}>0$ for every value of $w_{I}$, ensuring positive nodal thickness.

#### Locking treatments:

The shell formulation uses separate treatments for transverse-shear locking and the artificial thickness constraint associated with near-incompressibility. Transverse-shear locking becomes significant in the thin-shell limit, where standard displacement-based triangular elements develop artificially high shear stiffness. We reduce this locking using the MITC3 (Mixed Interpolation of Tensorial Components) assumed-strain interpolation of Lee and Bathe [17]. The covariant transverse-shear strains are sampled at the three edge midpoints and then re-interpolated over the element. This interpolation exactly reproduces constant shear fields on flat elements with parallel directors.

Near-incompressibility requires contraction of the body through its thickness when the in-plane area increases. A shell with an inextensible director cannot represent this thickness change and therefore constrains the transverse-normal strain. This can make the shell artificially stiff. The effect is particularly important for the nearly incompressible parameter values used with the Lee–Sacks model of Eq. (4).

The extensible director of Eq. (7) removes this restriction by allowing the director length to change. The through-thickness stretch is represented by the nodal field $\lambda_{t,I}$. For a flat element with parallel directors the corresponding transverse-normal Green–Lagrange strain reduces to

(11) $$E_{33}=\frac{1}{2}\left(\lambda_{t}^{2}-1\right).$$

For general director configurations, the strain is calculated from the complete interpolated director field in Eq. (8).

The strain energy is evaluated using three integration points in the plane of each triangle and two Gauss–Legendre points through the thickness. To represent additional variation through the thickness, we introduce one enhanced-assumed-strain (EAS) mode [33],

(12) $$E_{\zeta \zeta }^{\text{cov}}⟵E_{\zeta \zeta }^{\text{cov}}+\alpha_{e}\zeta ,$$

where $\alpha_{e}$ is an element-local parameter and ζ is the normalized through-thickness coordinate. The superscript cov denotes the covariant strain components in the shell’s natural coordinate system, which are subsequently mapped to Cartesian components. Because $\alpha_{e}$ is local to each element, it introduces no additional global nodal unknowns.

Together, the nodal thickness stretch and the element-local EAS parameter allow the transverse-normal stress to relax. Since $\alpha_{e}$ enters the potential only through the strain energy, stationarity with respect to $\alpha_{e}$ gives $\partial {\tilde{\Pi }}_{h}/\partial \alpha_{e}=0$. For a flat element, this corresponds to the first-moment condition

$$\int_{\Omega_{e}}S_{33}\zeta \;dV=0,$$

where $S_{33}$ is the transverse-normal second Piola–Kirchhoff stress and $\Omega_{e}$ is the reference element volume. Stationarity with respect to the nodal thickness-stretch parameters provides the complementary equations governing the mean thickness response. Consequently, the transverse-normal stress is relaxed in a variational sense rather than being set to zero at every integration point. This allows the full three-dimensional constitutive model, including its volumetric penalty, to be evaluated at the shell integration points.

#### Drilling stabilization:

Rotation about the shell director is called drilling rotation. It does not change the director direction and therefore has no stiffness in the director-based strain energy, producing a zero-energy mode. This mode is regularized using a penalty [25]. For the valve simulations, the penalty coefficient is

$$k_{d}=\gamma c_{0}t,\;\gamma =\frac{1}{2},$$

where $c_{0}$ is the ground-matrix shear modulus of the Lee–Sacks material, t is the shell thickness, and γ is a dimensionless penalty factor. The sum of these drilling penalties over all shell elements defines the regularization energy $\Pi_{d}$ appearing in Eq. (1). This is a small-rotation stabilization, and exact invariance of the penalty energy under finite rigid-body rotations is not assumed.

#### Internal fields and reduction:

The primary Newton iteration is formulated in terms of the nodal displacement and rotation variables U, while the through-thickness fields are updated separately. The nodal thickness variables w and element-local enhanced-strain parameters $\alpha ={\left(\alpha_{e}\right)}_{e}$ are collected into a=(w,α). The discrete potential is written as ${\tilde{\Pi }}_{h}(U,a)$. These internal fields enter the strain energy but not the contact or pressure-load terms. For fixed $U,a^{⋆}(U)$ denotes internal-variable values satisfying a stationarity condition: the derivatives of the potential energy with respect to these variables vanish, so infinitesimal variations produce no first-order change in energy. Substituting these values into the potential defines the reduced potential $\Pi_{h}(U)$,

(13) $$\frac{\partial {\tilde{\Pi }}_{h}}{\partial a}\left(U,a^{⋆}(U)\right)=0,\;\Pi_{h}(U)={\tilde{\Pi }}_{h}\left(U,a^{⋆}(U)\right).$$

The two internal fields are treated differently. The enhanced-strain parameter $\alpha_{e}$ is element-local, so its stationarity condition is one independent scalar equation per element. Each equation is solved locally. During the coupled final refinement, we use each element’s linearized equation to express the update to $\alpha_{e}$ in terms of the displacement, rotation, and thickness updates. We then substitute this expression into the remaining equations, preserving the coupling to $\alpha_{e}$. Thus, $\alpha_{e}$ does not enter the global system as an independent unknown. The thickness field w, however, is shared by neighboring elements. Its stationarity condition therefore forms a global sparse nonlinear system, which is solved using regularized inner Newton updates.

Eliminating the internal fields has an important consequence for differentiation. At an exactly stationary internal state with pressure forces held fixed, the chain rule applied to Eq. (13) gives

$$\frac{d\Pi_{h}}{\;dU}=\frac{\partial {\tilde{\Pi }}_{h}}{\partial U}+{\left(\frac{da^{⋆}}{dU}\right)}^{⊤}\frac{\partial {\tilde{\Pi }}_{h}}{\partial a}=\frac{\partial {\tilde{\Pi }}_{h}}{\partial U},$$

because $\partial {\tilde{\Pi }}_{h}/\partial a=0$ at $a^{⋆}$. At this state, the reduced residual can be obtained by differentiating ${\tilde{\Pi }}_{h}$ with the internal fields held fixed. The second derivative also includes the coupling arising from the dependence of $a^{⋆}$ on U. During the load path, a limited number of sequential updates is used for the internal fields. Therefore, α and w may not yet satisfy their stationarity conditions exactly during an intermediate iteration, and the primary residual is evaluated while holding their current values fixed. At the final load, a regularized refinement step accounts for the coupling among the three fields and verifies that the residuals associated with U,w, and α satisfy their prescribed tolerances, as described in Section 2.4.

#### Reported strain measures:

For interpretation, the tangential Green–Lagrange strain can be written to first order through the shell thickness as

(14) $$E_{tan}\left(\zeta \right)\approx E_{m}+\frac{t}{2}\zeta \kappa ,\;\zeta \in \left[-1,\;1\right],$$

where ζ is the normalized through-thickness coordinate, $E_{m}$ is the midsurface membrane strain, and κ is the bending strain coefficient. The subscript tan denotes the in-plane strain components. The complete finite-strain tensor, including higher-order through-thickness terms, is evaluated directly at the shell integration points and is not truncated using Eq. (14). Each strain tensor is represented by a scalar strain magnitude, defined using the norm,

$$\|E\|=\sqrt{E:E}={\left(\sum_{i,j}E_{ij}^{2}\right)}^{1/2}.$$

This norm combines all components of the strain tensor into a single scalar value and is invariant under orthonormal changes of coordinate frame. The membrane strain $\left\|E_{m}\right\|$ characterizes the in-plane deformation of the shell midsurface. The strain magnitudes on the upper and lower shell faces are approximated by

(15) $$\left\|E_{tan}(\zeta =\pm 1)\right\|\approx \left\|E_{m}\pm \frac{t}{2}\kappa \right\|,$$

which includes both membrane and bending contributions.

Unless stated otherwise, the reported shell strain is obtained by first taking the quadrature-weighted average of the full 3 × 3 Green–Lagrange strain tensor over the six integration points of each element: two through-thickness points at each of the three in-plane integration locations. The norm is then applied to the averaged tensor to obtain the reported scalar strain magnitude. For a flat element with a thickness-symmetric mapping, the equal weights at paired through-thickness points cancel the linear bending term and the EAS contribution $\alpha_{e}\zeta$ in the tensor average. On a curved element, however, the mapping from the shell coordinates to the Cartesian strain tensor varies through the thickness. Exact cancellation is therefore not assumed. Consequently, the reported tensor is a full three-dimensional element average that includes the transverse-normal strain and transverse-shear strains, as well as any remaining higher-order through-thickness contributions.

### 2.3. Contact and self-contact formulation

Contact is represented by a short-range repulsive surface energy, inspired by potential-based contact formulations [34, 8].

#### Distance measure and search:

Each surface triangle contributes one quadrature sample located at its centroid in the current configuration and weighted by its reference area $A_{s}$. This one-point rule approximates a surface integral over the reference surface, whose quadrature weights remain constant during deformation. For contact between separate shell surfaces, each sample uses a list of $N_{c}=6$ candidate target facets, identified using a KD-tree search based on the current facet centroids. The candidate lists are rebuilt after each accepted load step. Within a load step, the candidate set is fixed, but the facet that gives the minimum distance is re-evaluated from the current geometry during every contact-energy evaluation.

For each candidate facet $T_{k}$, the closest point $c_{k}$ is computed using barycentric coordinates located to the triangle face, edges, or vertices [35]. The corresponding regularized point-to-triangle distance is

(16) $${dist}_{\varepsilon }\left(x_{s},T_{k}\right)=\sqrt{{\left\|x_{s}-c_{k}\right\|}^{2}+\varepsilon_{d}^{2}},$$

where $x_{s}$ is the current position of the source-triangle centroid. The small value $\varepsilon_{d}=1\times 10^{-12}\;mm$ prevents an undefined derivative at exactly zero distance and has a negligible effect away from that point. The contact distance is the minimum over the candidate facets,

(17) $$d=\underset{k=1,\ldots ,N_{c}}{min}{dist}_{\varepsilon }\left(x_{s},T_{k}\right).$$

This distance accounts for closest points on facet edges and vertices, rather than only projections onto the facet plane.

#### Contact potential:

The unsigned distance d is measured between the shell midsurfaces. The contact-activation measure is

(18) $$g={\left\langle R_{\text{out}}-d\right\rangle }_{+},\;\langle x\rangle_{+}=max(x,0),$$

where $\langle x\rangle_{+}$ denotes the positive part of x and $R_{\text{out}}$ defines the short interaction range. The potential is inactive when $d\geq R_{\text{out}}$ and becomes active when the midsurface distance d falls below $R_{\text{out}}$. The interaction range is a numerical parameter and does not depend on the local thickness stretch in the present expression. The contact contribution to the total potential energy is,

(19) $$\Pi_{c}=\sum_{s}\pi_{s},\;\pi_{s}=k_{c}A_{s}g_{s}^{4},$$

where $k_{c}$ is the penalty coefficient, $A_{s}$ is the reference area associated with sample s, and the sum runs over the contact samples defined above. The quartic positive-part function ${\left\langle x\right\rangle }_{+}^{4}$ is three times continuously differentiable, with its value and first three derivatives vanishing at activation. Within a fixed closest-point branch, the contact kernel is $C^{3}$, its force is $C^{2}$, and its tangent is $C^{1}$. When one candidate facet is uniquely closest and the closest-point region is unchanged, automatic differentiation provides the derivative of that branch. With a fixed candidate list, the distance remains continuous, but switching the closest facet can produce a discontinuous gradient. The complete contact formulation is therefore piecewise smooth. Because the contact potential is part of the total energy, its branchwise residual and symmetric Hessian contributions are obtained automatically using the same differentiation procedure as for the strain energy.

#### Parameter continuation and self-contact:

For the valve simulations, the contact parameters are ramped linearly with the applied load fraction during the first half of the loading path and remain at their final values thereafter. The penalty coefficient increases from $k_{c}=1000\;kPa{\;mm}^{-3}$ to $8000\;kPa\;{mm}^{-3}$, while the interaction range decreases from $R_{\text{out}}=0.8\;mm$ to 0.5 mm. The wider initial range and lower penalty coefficient introduce repulsion gradually; reducing the range and increasing the coefficient then localize and strengthen the interaction. This numerical continuation assists nonlinear convergence. The same contact potential was used for self-contact in the pressurized-ring benchmark. For each source facet, candidate target facets were restricted to facets at least five circumferential cells away. This exclusion prevents spurious repulsion between nearby facets connected along the ring. In the patient-specific simulations, candidate target facets were restricted to the other leaflets; therefore, leaflet self-contact was not active.

### 2.4. Solver and implementation

The framework is implemented in JAX [16], which provides automatic differentiation, automatic vectorization of the element and contact kernels through vmap, just-in-time compilation, and execution on both CPUs and GPUs from a single code base. The full equilibrium problem comprises the primary and internal stationarity equations with pressure evaluated on the current geometry. Writing y=(U,w,α) and denoting a model or loading parameter by q, these equations can be collected as R(y,q)=0. The operators used to obtain corrections during load advancement and final refinement approximate the Jacobian of the full equilibrium equations, including the coupling among the primary and internal variables and the geometry dependence of the pressure forces. The following description distinguishes these correction operators from the full equilibrium Jacobian required for implicit solution sensitivities.

#### Derivatives and matrix-free operators:

During derivative evaluations for the primary Newton solve, the internal fields and pressure force vector are held fixed. Within a smooth contact branch, the discrete potential in Eq. (2) is a differentiable scalar function. Therefore, reverse-mode AD computes the residual as the gradient of this potential, $r=\partial {\tilde{\Pi }}_{h}/\partial U$, following the energy-based differentiation approach of the tatva framework [10]. The Hessian–vector product (Kv) is obtained by forward-over-reverse differentiation: a forward-mode directional derivative is applied to the reverse-mode residual in the direction v, giving

(20) $$Kv=\underset{\epsilon \to 0}{lim}\frac{r(U\;+\;\epsilon v)\;-\;r(U)}{\epsilon }.$$

Here, K=∂r/∂U is the tangent operator with the internal fields and pressure forces held fixed. AD evaluates this derivative using the chain rule, without finite-difference approximations. With the internal fields and pressure force vector held fixed, K is the Hessian of the total potential energy and is symmetric on each smooth contact branch. This statement concerns the fixed-field operator, rather than the Jacobian of the complete geometry-dependent equilibrium problem. The Krylov solve evaluates the primary tangent action without assembling that operator; auxiliary matrices are assembled for preconditioning and internal-field solves, as described below.

#### Internal fields and the applied operator:

During final equilibrium refinement, the element-local EAS updates are eliminated from the linearized equations. The remaining thickness-field coupling is included through the regularized reduced tangent

(21) $${\hat{K}}_{red}=K_{UU}^{\left(\alpha \right)}-K_{Uw}^{\left(\alpha \right)}{\left(K_{ww}^{\left(\alpha \right)}+\beta I\right)}^{-1}K_{wU}^{\left(\alpha \right)}.$$

The subscript red denotes reduction to the primary variables U, and the superscript (α) indicates that the EAS variables have been eliminated. The diagonal blocks describe the primary and thickness-field responses, while the off-diagonal blocks describe their coupling. The second term accounts for thickness-field changes in the primary correction. Here, I is the identity matrix, and β is $10^{-2}$ times the mean absolute diagonal entry of $K_{ww}^{\left(\alpha \right)}$. The inverse denotes a sparse linear solve and is not formed explicitly. The shift changes the Newton correction, not the equilibrium equations. The thickness-field residual is included in the reduced right-hand side, whereas the EAS residual is checked separately, as detailed in Table 1. The final correction therefore approximates the system obtained by eliminating both internal fields exactly.

#### Load stepping:

The applied pressure is increased incrementally, with each load step starting from the accepted configuration of the previous step. At step k, the pressure force vector is computed using the loaded-face geometry from $U^{\left(k-1\right)}$ and held fixed during the nonlinear solve. The work of the fixed pressure forces is therefore

(22) $$\Pi_{ext}^{\left(k\right)}(U)=F_{p}^{\left(k\right)}\cdot U,\;F_{p}^{\left(k\right)}=\int_{\Gamma_{p}(U^{\left(k-1\right)})}p^{\left(k\right)}N^{⊤}n\;da,$$

where $p^{\left(k\right)}$ is the applied pressure, N collects the element shape functions, and n and da are the face normal and area element of the frozen configuration. The load increment is adapted during loading and reduced when a step is rejected. At full load, the pressure forces are recalculated from the updated geometry, and the coupled equilibrium refinement is repeated.

#### Newton–Krylov solution:

Each load step is solved by an inexact Newton–Krylov method [36, 19], in which the linearized system

(23) (K+μI)ΔU=−r

is solved approximately and matrix-free using the preconditioned conjugate-gradient (CG) method. Here, μ>0 is the regularization parameter and I is the identity matrix. CG requires a symmetric positive-definite operator, whereas the shell tangent may become indefinite because of geometric nonlinearity and contact. To stabilize the correction in these cases, we add the diagonal shift μI, known as Levenberg regularization. Because the shifted operator can still be indefinite, CG checks the curvature of the local quadratic energy along each search direction. The iteration stops when this quantity is non-positive or nearly zero [37]. The returned correction is tested by a backtracking line search, which first tests the full update and reduces its step length if the potential-energy acceptance condition is not satisfied. If no acceptable step is found, the correction is rejected and μ is increased. Because the Levenberg shift modifies only the Newton correction and not the equilibrium equations, convergence is assessed using the original equilibrium residual r, so the final configuration must satisfy the original equilibrium equations to the prescribed tolerance.

#### Preconditioning:

To accelerate Krylov convergence when the linearized system contains large stiffness differences, we use a two-level preconditioner combining a positive diagonal (Jacobi) approximation D with a coarse correction:

(24) $$M^{-1}=D^{-1}+PA_{c}^{-1}P^{⊤}.$$

The diagonal term rescales local stiffness differences, while the coarse correction represents collective deformation across groups of nodes. Here, D includes internal-energy and approximate contact contributions, P maps coarse translations and rotations to the nodal variables, and $A_{c}$ is a regularized coarse approximation constructed from the assembled internal-energy tangent and approximate contact contributions. The coarse space uses three translational and three rotational rigid-body modes for each spatial group of nodes. The diagonal and coarse components both include the Levenberg shift from Eq. (23). The shift-dependent parts are updated at every Newton iteration, while the assembled internal-energy and contact contributions are refreshed every fourth iteration.

#### Load-path and convergence criteria:

The patient-specific simulations are quasi-static and introduce no inertia. A permissive residual tolerance is used to advance the load path, followed by stricter convergence checks at full load. Along the load path, the acceptance check after updating the internal variables is

$$\|r\|<5\times 10^{-2}\;max\left(\left\|{\bar{F}}_{p}\right\|,F_{0}\right),$$

where ${\bar{F}}_{p}$ is the nodal force corresponding to the target pressure, evaluated on the loaded-surface geometry held fixed during the load step, and $F_{0}=1\;mN$ prevents a vanishing load scale. At full load, the internal variables and geometry-dependent pressure force are updated, and the configuration is refined using the regularized reduced tangent in Eq. (21). The final static residual must satisfy

$$\|r\|\leq 10^{-3}\;max\left(\left\|F_{p}\right\|,F_{0}\right).$$

The internal-variable and pressure-force convergence checks must also hold at the same updated state. The pressure-force update criterion is defined in Section 3.5.4. If these criteria are not met within the prescribed maximum number of refinement iterations, the simulation is classified as unconverged. Table 1 summarizes the updates, with $r_{U}=r$ denoting the primary equilibrium residual.

## 3. Results

This section first presents verification of the constitutive, contact, and shell components, followed by differentiation verification and solver-performance comparisons. The formulation is then applied to three patient-specific aortic valves, including mesh and numerical-sensitivity checks.

### 3.1. Thick-walled cylinder inflation: constitutive-model verification

The isotropic neo-Hookean and Lee–Sacks laws and the anisotropic Holzapfel–Gasser–Ogden (HGO) model were verified using inflation of a thick-walled cylinder with an incompressible reference solution. A quarter cylinder with inner radius A=5mm and outer radius B=10mm was modeled under plane-strain conditions. Symmetry conditions were applied to the radial cut faces, and the outer wall was traction-free. The inner-wall loading was controlled by either prescribed radial displacement or pressure loading. Geometry and material parameters are summarized in Table 2. The two HGO fiber families lie in the circumferential–axial plane, oriented at $\beta =\pm 45^{\circ }$ to the circumferential direction:

$$a_{0}^{\left(1,2\right)}=cos\;\beta \;e_{\theta }\pm sin\;\beta \;e_{z}.$$

Their fiber invariant under plane strain is $I_{4}=\lambda_{\theta }^{2}\;{cos}^{2}\;\beta +{sin}^{2}\;\beta$. For the incompressible reference, the current radius r and principal stretches are

$$r(R)=\sqrt{R^{2}+a^{2}-A^{2}},\;\lambda_{\theta }=\frac{r}{R},\;\lambda_{r}=\frac{R}{r},\;\lambda_{z}=1,$$

where R is the reference radius and a is the deformed inner radius. The corresponding pressure is

$$P(a)=\int_{a}^{b_{d}}\frac{\sigma_{\theta \theta }\;-\;\sigma_{rr}}{r}\;dr,$$

where $b_{d}=r(B)$ is the deformed outer radius. This one-dimensional equilibrium integral provides a reference independent of the finite-element discretization. It was evaluated using 600 uniformly spaced points; increasing this number to 2400 changed the pressure by at most 0.00062%.

*In the displacement-controlled tests*, the inner radius a was prescribed and the resulting pressure was compared with P(a). The mean relative pressure errors were 0.05%, 0.15%, and 0.06% for the neo-Hookean, Lee–Sacks, and HGO models, respectively.

*In the pressure-controlled tests*, the computed inner radius differed from the reference by less than 0.05% over the tested range (Fig. 1b). At 15 kPa, the radial displacement profile agreed with the reference to within 0.01%, and the HGO fiber invariant agreed to within 0.04%. These results verify the three constitutive implementations with the near-incompressible plane-strain discretization.

### 3.2. Verification of the contact formulations

Three benchmarks examined different aspects of contact. Prescribed-motion analyses verified the quartic contact law and its numerical integration. A contact patch test examined force transmission using separate quadratic-penalty and augmented-Lagrangian formulations [38, 39]. A pressurized-ring benchmark then assessed coupled shell deformation and self-contact using the same quartic formulation as the valve simulations, with equilibrium obtained through direct energy minimization.

#### 3.2.1. Quartic contact formulation

The quartic contact law was examined through traction verification, surface-integration accuracy, and the contact response to changes in the closest target triangle. A rigid 4 × 4 mm source surface was centered below a fixed 10 × 10 mm target surface (Fig. 2a). The target had a slope of 0.02 in the x direction, and the initial central vertical separation was $d_{0}=0.30\;mm$. The source underwent a prescribed displacement s in the z direction. Both surfaces were triangulated. For each source-triangle centroid, d was the shortest distance to the closest of six candidate target triangles. Because the target was tilted, this local distance varied across the source surface. Differentiating the quartic energy in Eq. (19) gives the analytical traction magnitude during active contact,

$$\sigma =4k_{c}g^{3},\;g={\left\langle R_{\text{out}}-d\right\rangle }_{+}.$$

The contact parameters were $k_{c}=8000{\;kPa\;mm}^{-3}$ and $R_{\text{out}}=0.5\;mm$. The AD derivative of the quartic law agreed with this analytical expression, with a maximum relative error of 2.2 × 10^−16^ (Fig. 2b).

To verify the numerical integration of contact energy and force over the source surface, the source mesh was refined while keeping the tilted target geometry fixed. At each refinement step, the source facet size was reduced to half its previous value. The computed contact energy and total contact force were compared with analytical surface integrals. Both relative errors decreased by approximately a factor of four at each refinement step. Thus, the centroid-based integration approached the analytical energy and force with second-order accuracy for this geometry.

A separate geometric test used a single moving source point and two fixed, non-deforming target triangles. The triangles represented two separated regions of the target surface, with a gap between them. The source point moved horizontally above them along $x_{p}(q)=(q,\;0,\;0)$, where q denotes its horizontal position. The test examined whether the contact force remained continuous when the nearest target changed from one triangle to the other. The contact distance was

$$d(q)=min\left(d_{1}(q),d_{2}(q)\right),$$

where $d_{1}(q)$ and $d_{2}(q)$ are the shortest distances from the source point to the first and second target triangles, respectively. Using a reference area of 1 mm^2^ for the source point, Fig. 2c plots $\partial \Pi_{c}/\partial q$, which measures how the contact energy changes as the source point moves. The horizontal contact force on the point is the negative of this derivative:

$$F_{x}=-\frac{\partial \Pi_{c}}{\partial q}.$$

At q≈−0.0266mm, the nearest target changed from one triangle to the other. The contact energy remained continuous, but the horizontal force changed abruptly. This shows that choosing the nearest target triangle can introduce force discontinuities even when the quartic contact law itself is smooth.

#### 3.2.2. Contact patch test

Uniform force transmission was examined using two 10 mm×10 mm×5 mm neo-Hookean blocks placed one above the other to form a 10 mm cube (Fig. 3a). The material parameters were E=100kPa and ν=0.30. A quarter-symmetry tetrahedral mesh was used. Compression was imposed by incrementally prescribing a downward displacement of the upper block’s top surface, reaching 3 mm (30% nominal compression). The bottom surface of the lower block was constrained in the vertical direction and free to slide laterally. Symmetry conditions were applied to the cut faces, and exterior lateral surfaces were traction-free. The contact surfaces were frictionless and independently meshed.

Three configurations were compared: a single block without a contact interface, two blocks with quadratic-penalty contact (κ=1000), and two blocks with augmented-Lagrangian contact using the same penalty and 12 augmentation passes. The single-block model provided the numerical reference, while the analytical solution was a uniform uniaxial-stress state. Among the three configurations, the quadratic-penalty solution had the largest force error. It differed from the analytical compressive force by at most 0.09% over the prescribed displacement range (Fig. 3b). At the final compression, the single-block and augmented-Lagrangian solutions matched the analytical strain $E_{33}=-0.2550$ to the reported precision; the quadratic-penalty relative strain error was approximately 0.06%. In all three simulations, the element-wise axial strain was nearly uniform, with a standard deviation of order 10^−8^. Thus, the axial strain was nearly constant throughout the blocks, consistent with the uniform analytical deformation.

#### 3.2.3. Self-contact of a pressurized ring

Self-contact was assessed on a circular ring using the same shell element and quartic point-to-triangle contact formulation as in the valve simulations. The ring had radius R=10mm and thickness t=1.2mm. Its isotropic strain-energy density was

$$\psi (E)=\frac{\lambda }{2}(tr\;E{)}^{2}+\mu E:E,$$

where E is the Green–Lagrange strain tensor. Young’s modulus E=100kPa and Poisson’s ratio ν=0 give λ=0 and μ=50kPa. Setting ν=0 removes Poisson coupling to facilitate comparison with the two-dimensional reference. The shell was discretized using 120 triangular elements. A 0.5% radial imperfection made the initial ring slightly oval and guided the buckling pattern. The out-of-plane displacement was constrained by $u_{z}=0$, while in-plane motion remained unconstrained. Equilibrium solutions for this benchmark were obtained by direct energy minimization up to p=144Pa.

An independent two-dimensional reference used 120 straight segments to approximate the continuum theory of an inextensible ring under external pressure [40, 41]. The reference was formulated per unit out-of-plane width, with $I=t^{3}/12$. Its continuum bending and pressure contributions to the potential were

(25) $$\Pi_{e}=\frac{EI}{2}\int_{0}^{L}{\left(\kappa -\kappa_{0}\right)}^{2}\;ds+pA,\;L=2\pi R,$$

where κ and $\kappa_{0}=1/R$ are the current and initial centerline curvatures, and A is the enclosed area. In the segmented model, changes in segment angles determined the bending energy, a segment-length penalty maintained near inextensibility, and a quadratic node-pair penalty activated contact at separations below t. This reference therefore provides a comparison independent of the shell and quartic contact implementations.

The nearly circular shell evolved into an oval and then formed the expected two-lobe self-contact configuration [42, 41]. The ring separation d was defined as the minimum distance between initially opposite points on the ring centerline, as plotted in Fig. 4c. At the final load, d=1.20mm, approximately 0.37% below the reference separation t=1.2mm. An independent triangle-intersection check detected no intersections between non-adjacent midsurface triangles. The shell and two-dimensional reference differed by approximately 0.05% in ovalization, $\left(r_{\text{max}}-r_{\text{min}}\right)/R$, and 0.09% in ring separation d. Their final circumferences were respectively 0.88% and 0.02% below 2πR, reflecting the greater circumferential contraction permitted by the shell.

### 3.3. Moment-loaded roll-up: large-rotation shell bending

The three-node MITC3 seven-parameter extensible-director shell was evaluated under large rotation using the moment-loaded roll-up benchmark [43, 32]. A clamped strip of length L=12mm, width b=1mm and thickness t=0.1mm was loaded by an end moment. The material was St Venant–Kirchhoff, with E=1.2GPa and ν=0. Motion was restricted to the x–z plane. Setting ν=0 removes Poisson-induced transverse curvature, permitting comparison with analytical pure bending. With $I=bt^{3}/12$, the bending stiffness was $EI\;=\;0.1\;N\;mm^{2}$. The thickness stretch was fixed at the clamped edge and allowed to vary at all other nodes. The EAS mode was disabled. This benchmark therefore assesses the bending response without EAS relaxation; the formulation with EAS is assessed using plate inflation (Section 3.4).

The analytical solution is a circular arc with uniform curvature κ=M/EI. Defining the total rotation as ϕ=ML/EI, the tip coordinates are

$$\frac{x_{\text{tip}}}{L}=\frac{sin\;\phi }{\phi },\;\frac{z_{\text{tip}}}{L}=\frac{1\;-\;cos\;\phi }{\phi }$$

The strip forms a complete circle at M=2πEI/L=52.4mNmm.

In the small-moment limit, the shell reproduced the analytical cantilever deflection $z_{\text{tip}}=ML^{2}/(2EI)$ with a relative error of 3.3 × 10^−4^%. For 32, 64 and 128 triangular elements, the maximum normalized tip errors were 9.03%, 0.99% and 0.05%, respectively, compared with the analytical solution. In each case, the maximum occurred at complete roll-up. With 128 elements, the mean error over the loading ramp was 0.02%, and the final closure gap between the tip and the clamped origin was 6.56 × 10^−3^ mm. The tip-displacement curves also followed the analytical response (Fig. 5c). The transverse displacement reached approximately 8.7 mm before returning close to zero. During roll-up, the tip temporarily moved past the clamped origin in the x direction, giving $x_{\text{tip}}<0$. It then returned near the origin as the circle closed, reproducing the analytical reversal of tip motion under large rotation.

### 3.4. Circular-plate inflation: shell response and mesh sensitivity

Circular-plate inflation examined the combined membrane and bending response under large deformation. A circular plate with R=7.5mm and t=0.5mm was inflated by uniform pressure to 35 kPa. At every boundary node, displacement was constrained in all three directions, while director rotations, nodal thickness stretch, and element-local enhanced-strain variables remained free. The material used an isochoric Mooney–Rivlin model with $C_{1}=80\;kPa$, $C_{2}=20\;kPa$ and a volumetric penalty $\frac{1}{2}K(ln\;J{)}^{2}$, where K/μ=50 and $\mu =2\left(C_{1}+C_{2}\right)$. Five uniformly refined meshes contained 19, 61, 217, 817 and 3169 nodes, with the mean element size reduced to approximately half its previous value at each refinement level. The final apex displacement increased monotonically from 7.45 to 9.69 mm. The finest-mesh value was 0.92% higher than the preceding mesh level. On the fourth mesh level, increasing the pressure ramp from 24 to 32 steps changed the final apex displacement by 0.0022 mm (0.02%).

Figure 6a compares the fourth-level 32-step response with the Cirak–Ortiz subdivision-shell and Hughes–Carnoy axisymmetric-solid results, both obtained from Fig. 8 of Cirak and Ortiz [44] [45]. At 35 kPa, the computed apex displacement was9.59 mm, compared with approximately 9.03 mm and 9.10 mm, respectively: 6.3% and 5.4% higher. The published models used different formulations, so these differences cannot be attributed to discretization error alone. Incompressibility was enforced directly by Cirak and Ortiz and through a penalty by Hughes and Carnoy. On the fourth-level mesh, increasing K/μ from 50 to 1000 reduced the apex displacement by 11.9%.

#### 3.4.1. Differentiability verification

Differentiability of the converged response was verified using the circular plate at p=20kPa. On the second mesh level, the relative residual was 3.2 × 10^−13^, and the tangent for the unconstrained degrees of freedom was positive definite. Derivatives of the converged apex displacement W with respect to $C_{1}$, $C_{2}$, thickness t, and pressure p were obtained by differentiating the equilibrium equations. These implicit derivatives, $dW/dC_{1},\;dW/dC_{2},\;dW/dt$, and dW/dp, were compared with central finite differences from independent nonlinear re-solves. For the finite-difference calculations, each parameter was varied separately using 22 relative perturbation sizes between 10^−1^ and 10^−8^. The bulk modulus and drilling-penalty coefficient were held fixed during these perturbations. For a differentiable equilibrium branch with nonsingular $R_{y}$, implicit differentiation of the complete equations R(y,q)=0 gives

$$\frac{dy}{\;dq}=-R_{y}^{-1}R_{q}.$$

The Jacobian in this relation is that of the equilibrium equations, including internal-field coupling and the geometry dependence of the pressure force. It is distinct from a regularized correction operator used to reach equilibrium.

As the perturbation size decreased, the relative errors between implicit and central finite-difference apex-displacement derivatives initially decreased approximately quadratically, as expected for central finite differences. At very small perturbations, the finite-difference calculation subtracts nearly equal displacements and divides by a very small parameter increment. This amplifies round-off and nonlinear-solve errors, causing the derivative errors to increase (Fig. 6b). For each parameter, the derivative of the full equilibrium solution agreed with finite differences, with a minimum relative error of 3.4 × 10^−10^ or smaller over the tested perturbation sizes. Similar agreement was obtained on the next mesh level. These results verify implicit solution derivatives for the tested contact-free shell equilibrium solutions. The quartic contact-energy derivatives were tested separately in Section 3.2.

#### 3.4.2. Computational performance

The circular-plate benchmark was used to compare CPU and GPU execution of the same matrix-free solver using a 32-step pressure ramp to 35 kPa. Levels L2–L4 contained 451, 1759, and 6967 free degrees of freedom, respectively, including nodal variables and one enhanced-strain variable per element. The solver evaluated Hessian–vector products using AD and Jacobi-preconditioned CG.

CPU and GPU runs were performed on the same compute node using 12 physical Intel Xeon Gold 5317 cores and one NVIDIA A100-SXM4-40GB, respectively. All runs used float64 arithmetic with Python 3.12.3, JAX/jaxlib 0.10.2, NumPy 2.4.2, and SciPy 1.17.0. Five independent timed runs were performed for each device and mesh level, giving 30 timed runs. Solve times include first-use JIT compilation and nonlinear and linear solution, but exclude imports and mesh generation. Convergence was checked using the implemented residual indicator,

$$\rho =\frac{\left\|g\right\|}{max(1,\;\|f\|)},$$

where g is the numerical energy-gradient vector on the unknown variables and f is the numerical pressure-load vector evaluated at the start of each load step. Because the unknowns include translations, rotations, and internal variables, this indicator uses the benchmark’s fixed mm–kPa coordinate convention. All 30 timed runs satisfied $\rho \leq 10^{-6}$ at every load step and produced final apex displacements agreeing to the reported precision: 8.46, 9.32, and 9.59 mm for L2–L4, respectively.

Median CPU solve times were 42.59, 238.16, and 1383.00 s, compared with 32.94, 56.42, and 96.25 s on the GPU for L2–L4, respectively (Fig. 6c). The corresponding ratios of CPU to GPU median times were 1.29, 4.22, and 14.37. These speedups are relative to the specified 12-core CPU baseline on the tested hardware. Recorded Krylov iteration counts were identical across CPU repeats but varied by approximately 10–13% across GPU repeats. The timing ratios therefore reflect differences in numerical work as well as execution speed.

### 3.5. Application: patient-specific aortic valve simulations

The formulation was applied to three patient-specific aortic valves: two tricuspid and one bicuspid. All cases used the same Lee–Sacks parameters and solver, with patient-specific geometry and annulus motion.

#### 3.5.1. Geometry, discretization and boundary conditions

Leaflet geometries were reconstructed from manual segmentations in the open reference configuration, following our previous workflow [28]. The open reference configuration was assumed to be stress-free. The collection and analysis of the clinical imaging data were approved by the Institutional Review Boards at the Children’s Hospital of Philadelphia and the University of Pennsylvania. Medial surfaces were extracted using an interactive skeletonization tool in 3D Slicer [46, 47], smoothed while preserving the attachment boundaries and free edges, and discretized with triangular shell elements. ACVD remeshing was used where needed [48]. All leaflets had a uniform thickness of 1.2 mm. The nominal thickness follows the previous modeling workflow [28], informed by the autopsy study of Sahasakul et al. [49]. It was not measured separately for these patients. A uniform pressure load was applied to the ventricular face and ramped to 10 kPa, approximately 75 mmHg. Prescribed annulus displacements were obtained from image registration [28]. The mean annulus displacement was subtracted from each annulus nodal displacement to remove overall translation while retaining relative annulus motion. These displacements were ramped with the pressure.

#### 3.5.2. Leaflet strain, stress and computational performance

All three CPU simulations reached 10 kPa. Patients 1 and 2 completed 40 load steps without rejection; patient 3 completed 45 accepted steps after one rejected attempt. The closed configurations are shown in Fig. 7. After final internal-field refinement and recalculation of the pressure force from the deformed geometry, the relative equilibrium residuals $\|r\|/\left\|F_{p}\right\|$ were below 10^−4^ in all three cases, satisfying the convergence criterion of 10^−3^.

The median Green–Lagrange strain magnitudes (‖E‖) were 0.19, 0.23, and 0.21 for patients 1–3, respectively. Higher strains occurred near the commissures and the free edges involved in coaptation. The maximum strain magnitudes were 0.373, 0.362, and 0.450, respectively. Maximum principal Cauchy stress ($\sigma_{1}$) was estimated by evaluating the constitutive response at each element’s averaged Green–Lagrange strain tensor. Median stresses were 81.0 kPa, 101.2 kPa, and 108.8 kPa for patients 1–3, respectively. The maximum stress were 222.9 kPa, 191.4 kPa, and 494.5 kPa. GPU execution reduced the median runtime by factors of 5.79, 5.27, and 6.52 for patients 1–3, respectively, on the tested hardware. Table 3 summarizes the mechanical results and CPU–GPU performance, while Fig. 7 shows the reference and closed configurations with the strain and stress fields.

#### 3.5.3. Mesh-sensitivity study

For each patient, closure was repeated on independently generated ACVD meshes [48]. Of 24 simulations, 22 reached 10 kPa, completed refinement without an unrecovered stall, and satisfied $\|r\|/\left\|F_{p}\right\|\leq 10^{-3}$. The patient 2 mesh with 2800 dof stopped at 2.99 kPa. The patient 3 mesh with 10500 dof reached the target pressure but was excluded because its final relative residual was 4.08 × 10^−3^. Three quantities were examined: median Green–Lagrange strain magnitude, root-mean-square nodal displacement, and reference-area-weighted mean contact pressure over the active contact region (Fig. 8). Maximum strain was excluded because its discrete value is sensitive to local refinement near the constrained commissures.

For each patient, we selected the nearest accepted meshes with fewer and more dof than the mesh reported in Table 3. Each quantity on these meshes was compared with its value on the patient’s finest accepted mesh. For each quantity, the larger absolute relative difference from the two neighboring meshes was reported as a percentage of the finest-mesh value. The differences for patients 1–3 were 0.43%, 0.17%, and 1.15% for median strain; 0.32%, 1.32%, and 1.31% for root-mean-square nodal displacement; and 6.00%, 5.65%, and 6.16% for mean contact pressure. Median strain and displacement therefore showed limited mesh sensitivity near the selected resolutions, while contact pressure was more sensitive. Because the meshes were generated independently, changes with increasing dof were not necessarily monotonic.

#### 3.5.4. Numerical-sensitivity checks

Additional simulations examined sensitivity to the pressure-force update tolerance and through-thickness quadrature. At full load, pressure-force updates stopped when,

$$\frac{\left\|F_{p}^{\left(j\right)}\;-\;F_{p}^{\left(j-1\right)}\right\|}{\left\|F_{p}^{\left(j\right)}\right\|}\leq \varepsilon_{p},$$

where j is the update number and $\varepsilon_{p}$ is the pressure-force tolerance. For patient 2, the simulation was repeated with $\varepsilon_{p}=10^{-5},10^{-4}$, and 10^−3^. Relative to the 10^−4^ case, the monitored strain, displacement, and contact-distance measures changed by at most 0.008%. The reported simulations used $\varepsilon_{p}=10^{-4}$, which required 10 pressure-force updates in this test, compared with 14 for 10^−5^. The looser tolerance of 10^−3^ produced a relative equilibrium residual of 8.90×10^−4^, close to the reporting threshold of 10^−3^. In all three patient models, the standard two-point Gauss rule through the thickness was compared with four-point Gauss and five-point Lobatto rules, retaining three in-plane integration locations. The Lobatto rule also samples the shell faces. Relative to the two-point rule, the largest changes across the three patients were 0.82% in median Green–Lagrange strain magnitude, 0.59% in nodal displacement magnitude, and 0.10% in the minimum distance from a source-triangle centroid to an opposing triangle. These checks showed limited sensitivity of the monitored mechanical quantities to the tested pressure-force tolerances and quadrature rules.

## 4. Discussion

We developed shell and contact implementations for nonlinear valve mechanics using computational building blocks from tatva’s energy-based AD framework [10]. We compared constitutive and shell responses with reference solutions [43, 32, 44, 45] and checked contact-force calculations in dedicated benchmarks. Contact-free sensitivity analyses further showed agreement between derivatives of converged shell solutions and finite-difference estimates. The implementation supported nonlinear forward simulation of three patient-specific aortic valves with tricuspid and bicuspid geometries, and the matrix-free solver achieved shorter runtimes on the GPU than on the CPU.

### 4.1. Patient-specific valve simulations and numerical sensitivity

All three baseline valve simulations reached 10 kPa, with final relative residuals below 10^−4^ for the original, unregularized equilibrium equations. Median Green–Lagrange strain magnitudes were approximately 19%, 23%, and 21% for patients 1–3, respectively (Table 3), with higher magnitudes near the commissures and contacting free edges. This spatial pattern was qualitatively consistent with observations from our previous registration study [28]. That study reported leaflet-type cohort means of 17.3%–17.7% for tricuspid valves and 11.9%–16.5% for bicuspid valves [28]. Jermihov et al. reported maximum in-plane logarithmic strains of 15.7% for the tricuspid valve and 18.1%–25.5% across bicuspid geometries using their baseline material parameters [50]. Differences in strain estimates, patient populations, geometry, and loading limit direct quantitative comparison among these studies.

Mean contact pressure was more sensitive to mesh resolution than median strain and root-mean-square displacement (Section 3.5.3), suggesting that mesh selection should account for the mechanical quantity of interest. For the accepted meshes neighboring the reported resolutions, the largest relative differences from each patient’s finest accepted mesh were 1.15% in median strain, 1.32% in root-mean-square displacement, and 6.16% in mean contact pressure. Because the meshes were generated independently, these differences reflect changes in both resolution and mesh distribution. Additional checks showed that the strain, displacement, and contact-distance measures varied only slightly with the tested pressure-force update tolerances and through-thickness quadrature rules (Section 3.5.4). For patient 2, the selected 10^−4^ pressure-force tolerance provided a balance between computational effort and equilibrium accuracy. Tightening it to 10^−5^ increased the number of updates, while loosening it to 10^−3^ increased the final equilibrium residual.

### 4.2. Differentiability and computational performance

For the contact-free plate, implicit derivatives of the converged apex displacement agreed with finite differences from independent nonlinear re-solves for all four tested parameters (Section 3.4.1). The contact tests separately checked contact-energy derivatives within fixed contact branches (Section 3.2). Table 4 summarizes how differentiation is used in related biomechanical studies. Adjoint methods [51] and PyTorch-FEA [15] support parameter estimation, and energy-trained NNFE models provide rapid predictions after training [11]. A trained network can remain differentiable with respect to its inputs, but those derivatives describe the learned approximation and require their own verification. The present implementation focuses on shell-based forward valve simulation, combining large-deformation leaflet mechanics and contact with matrix-free computation on CPUs and GPUs. Its use of extensible-director shells distinguishes the mechanical discretization from the solid-element approaches listed in the table.

The CPU–GPU comparisons demonstrate acceleration of the reported matrix-free implementations on the tested hardware. For circular-plate inflation, the ratio of CPU to GPU median solve times increased from 1.29 at L2 to 14.37 at L4 (Fig. 6(c)). For the patient-specific valves, the ratios of CPU to GPU median runtimes were 5.27–6.52 (Table 3). These ratios are relative to the specified 12-core CPU baseline. The plate solve times include first-use JIT compilation, and their ratios reflect differences in Krylov iteration counts as well as execution speed. Tatva supports hybrid workflows by allowing JAX-based computations to be combined with external solvers [10]. The valve speedups were achieved with a hybrid workflow that combines GPU computation with contact searches, some auxiliary solves, and host-side solver operations on the CPU.

### 4.3. Limitations and future work

Contact and internal shell fields introduce additional computational requirements. Tatva provides matrix-free tangent–vector products and coloring-based sparse tangent reconstruction from the same AD-derived residual [10]. Coloring requires a sparsity pattern covering both shell and contact couplings. As contact candidates change, the pattern must be updated or kept broad enough to cover possible new interactions beyond the shell-mesh connectivity. Furthermore, during final valve refinement, global elimination of the nodal thickness variables can make the reduced tangent more densely coupled than the original mixed system (Eq. (21)). Our regularized, matrix-free Newton–Krylov solver accounts for internal-field coupling without explicitly constructing the complete reduced tangent. Auxiliary matrices are retained for preconditioning and internal-field updates. Coloring remains a viable alternative when the coupling pattern is represented correctly; its relative efficiency for the coupled valve problem would require a matched comparison of complete solves. JAX execution also involves memory and compilation considerations. Reverse-mode AD stores intermediate quantities for derivative evaluation [53]. For contact calculations, fixed-size candidate arrays with unused entries ignored can avoid dimension changes that would require recompilation. These arrays can still contain changing contact pairs, whose couplings must be covered by any coloring pattern. Changing the closest facet within an existing array does not itself require recompilation, but can introduce nonsmooth contact forces (Section 3.2). Further runtime reductions may be possible by moving selected CPU operations to the GPU and improving preconditioning to reduce the number of Krylov iterations [19].

In the valve simulations, although the geometries and prescribed annulus motion are patient-specific, the simulations use isotropic Lee–Sacks parameters found in the literature, uniform thickness, and an open reference state assumed to be stress-free. Representing regional thickness variation in the shell formulation would account for spatial differences in membrane and bending stiffness that can influence leaflet deformation and coaptation. Within the current JAX implementation, structurally motivated anisotropic constitutive models, such as the octahedral fibrous model of Parvez et al. [54], could represent the effects of fiber organization and pre-stretch in the valve simulations. Future work can build on the derivative checks to verify sensitivities of the converged valve solutions with active contact. Coupling the JAX-based forward solver with inverse parameter-estimation methods could further improve patient-specific simulations by estimating constitutive parameters from imaging-derived leaflet motion. The physics-informed neural network approach of Wu et al. [55] demonstrates the potential of image-informed material estimation for this purpose. Integrating the present implementation with machine-learning surrogates, such as the physics-conditioned neural operator developed by Koohy et al. [56], could accelerate repeated response evaluations within these inverse analyses.

## 5. Conclusion

This work demonstrates GPU-accelerated, shell-based simulation of patient-specific valve mechanics with contact within an AD-based framework. The benchmarks provided verification evidence for the tested components and selected coupled problems, including equilibrium sensitivities for contact-free plates. For the valve models considered, the matrix-free solver achieved 5.3–6.5-fold speedups on the GPU relative to corresponding CPU runs on the tested hardware. These reductions in solution time make repeated analyses of how geometry, constitutive parameters, and loading influence valve function more practical. The implementation provides a foundation for future gradient-based inverse studies and machine-learning coupling.

## Figures and Tables

**Figure 1:**
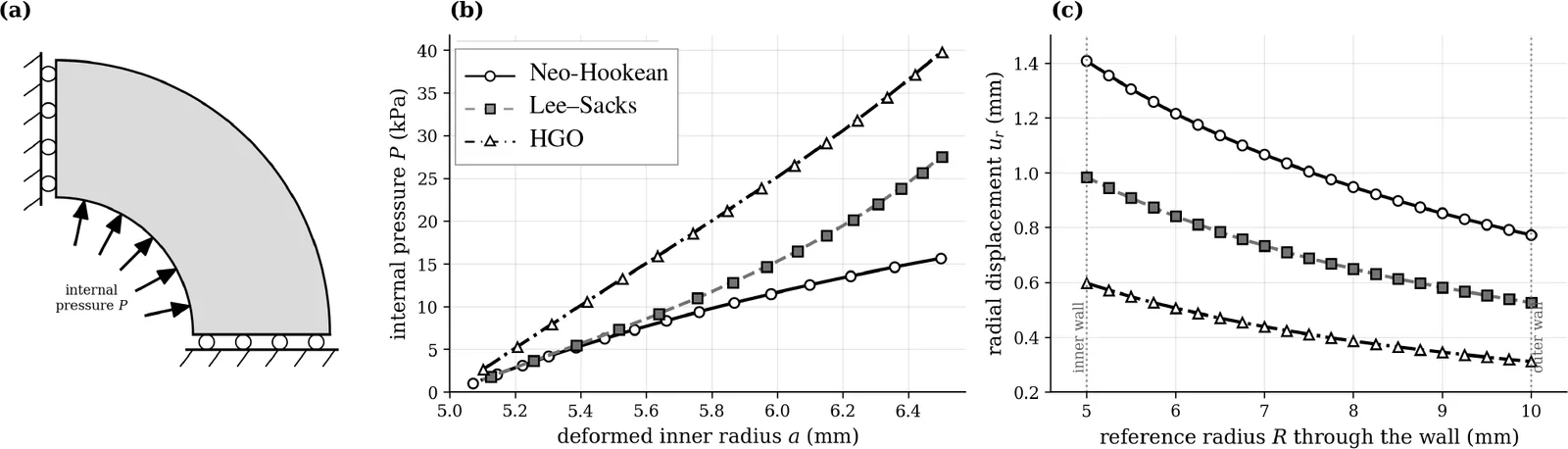
Finite-element results are compared with the incompressible reference solutions for thick-walled cylinder inflation. (a) Boundary conditions; (b) internal pressure versus deformed inner radius; and (c) radial displacement through the wall. Lines show reference solutions and markers show finite-element results.

**Figure 2:**
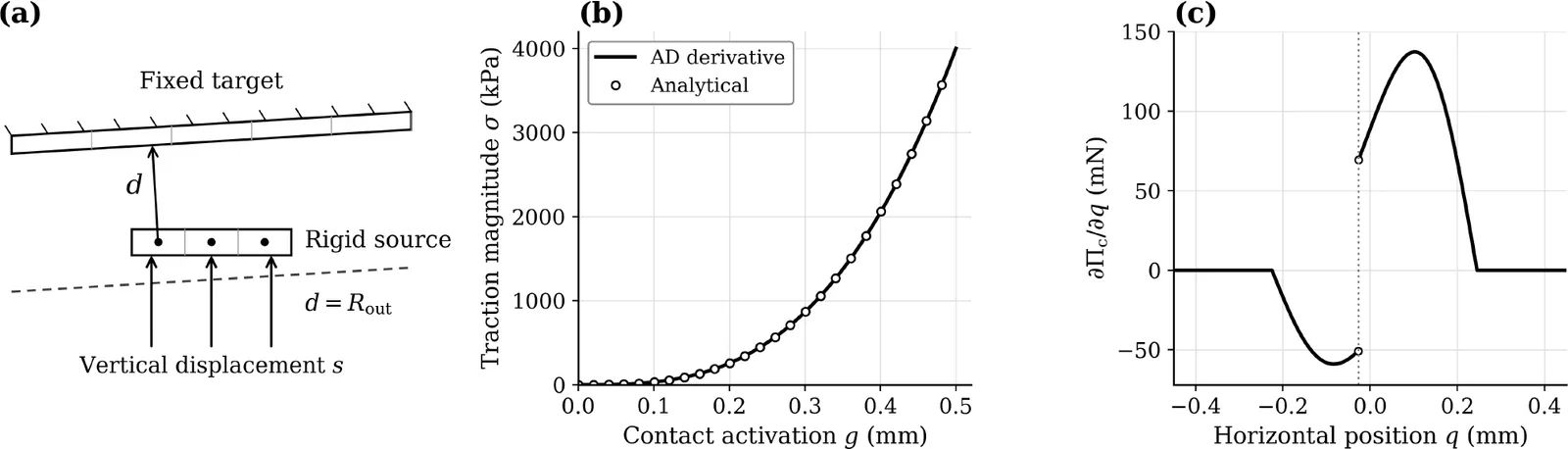
Verification of the quartic contact formulation. (a) Schematic of the contact configuration; (b) analytical and AD traction magnitudes; and (c) contact-energy gradient $\partial \Pi_{c}/\partial q$ versus horizontal source-point position q in the separate geometric test. The dotted line marks the change in the closest target triangle.

**Figure 3:**
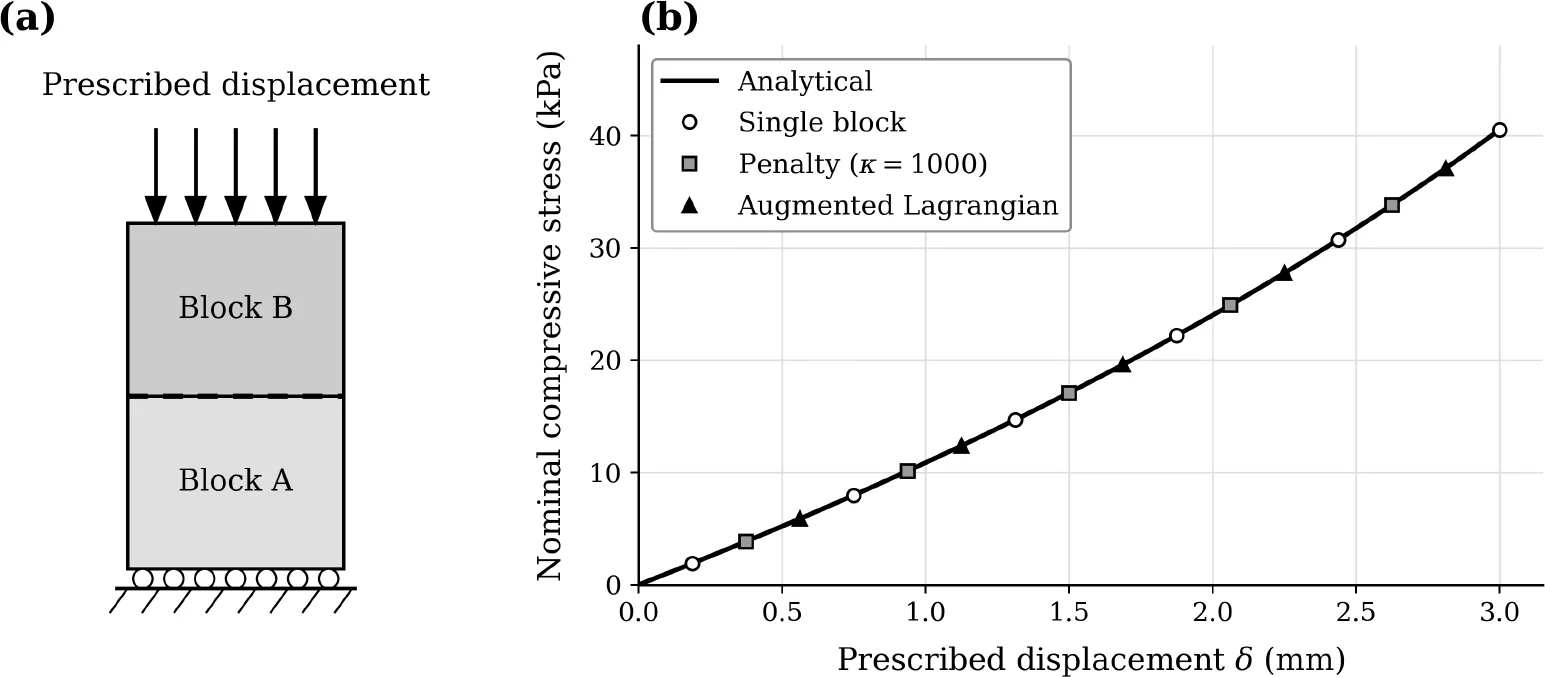
Contact patch test under displacement-controlled compression: (a) geometry and boundary conditions; and (b) nominal compressive stress versus prescribed downward displacement for the analytical, single-block, quadratic-penalty and augmented-Lagrangian solutions.

**Figure 4:**
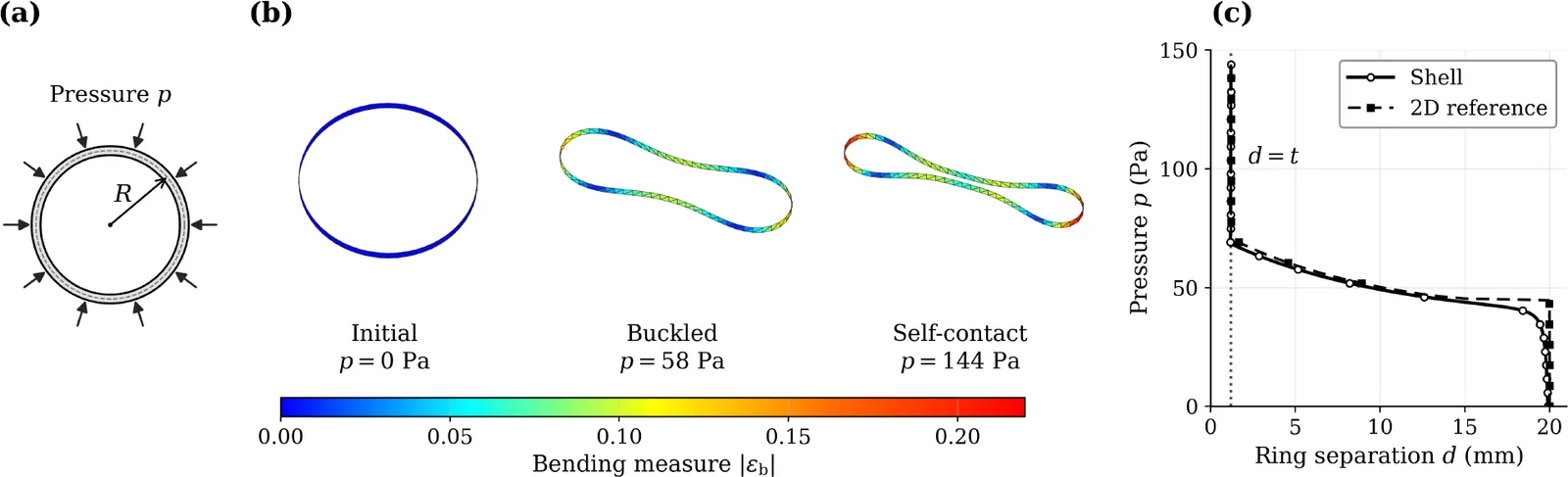
Self-contact of a shell ring under uniform external pressure. (a) Geometry and boundary conditions; (b) initial, buckled and self-contact shell midsurfaces colored by the curvature-based bending measure $\left|\varepsilon_{b}\right|=(t/2)\left|\kappa -\kappa_{0}\right|$, where κ and $\kappa_{0}$ are the current and initial centerline curvatures; and (c) pressure versus ring separation d for the shell and the two-dimensional segmented-ring reference.

**Figure 5:**
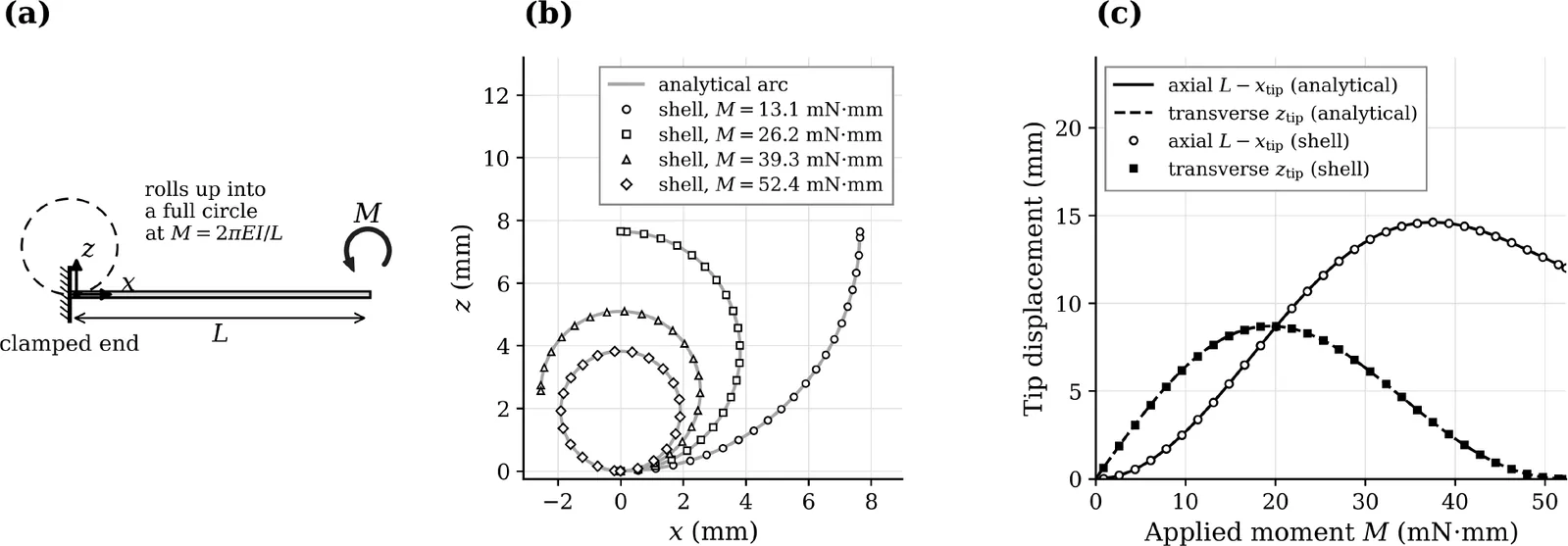
Moment-loaded roll-up. (a) Geometry and boundary conditions; (b) deformed shell midline at four moment levels compared with the analytical circular arcs; and (c) axial $L-x_{\text{tip}}$ and transverse $z_{\text{tip}}$ tip displacements versus applied moment.

**Figure 6:**
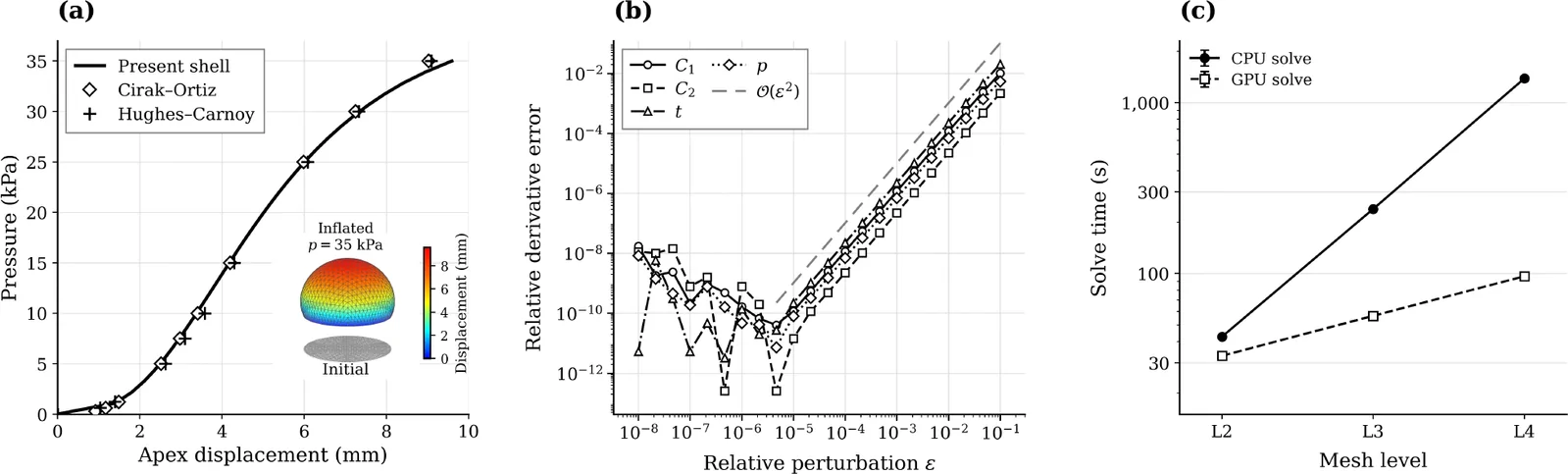
Circular-plate inflation and derivative verification. (a) Pressure–apex displacement response on the fourth mesh (817 nodes; 32 steps), compared with Cirak–Ortiz and Hughes–Carnoy results with initial and inflated configurations at 35 kPa. (b) Relative errors between implicit and central finite-difference apex-displacement derivatives for $C_{1}$, $C_{2}$, t, and p versus perturbation size ε at 20 kPa. The gray dashed line indicates second-order convergence. (c) CPU–GPU solve times for circular-plate inflation using 32 pressure steps to 35 kPa. Points show medians of five runs. The time axis is logarithmic. CPU: 12 Xeon Gold 5317 cores; GPU: one NVIDIA A100-SXM4-40GB.

**Figure 7:**
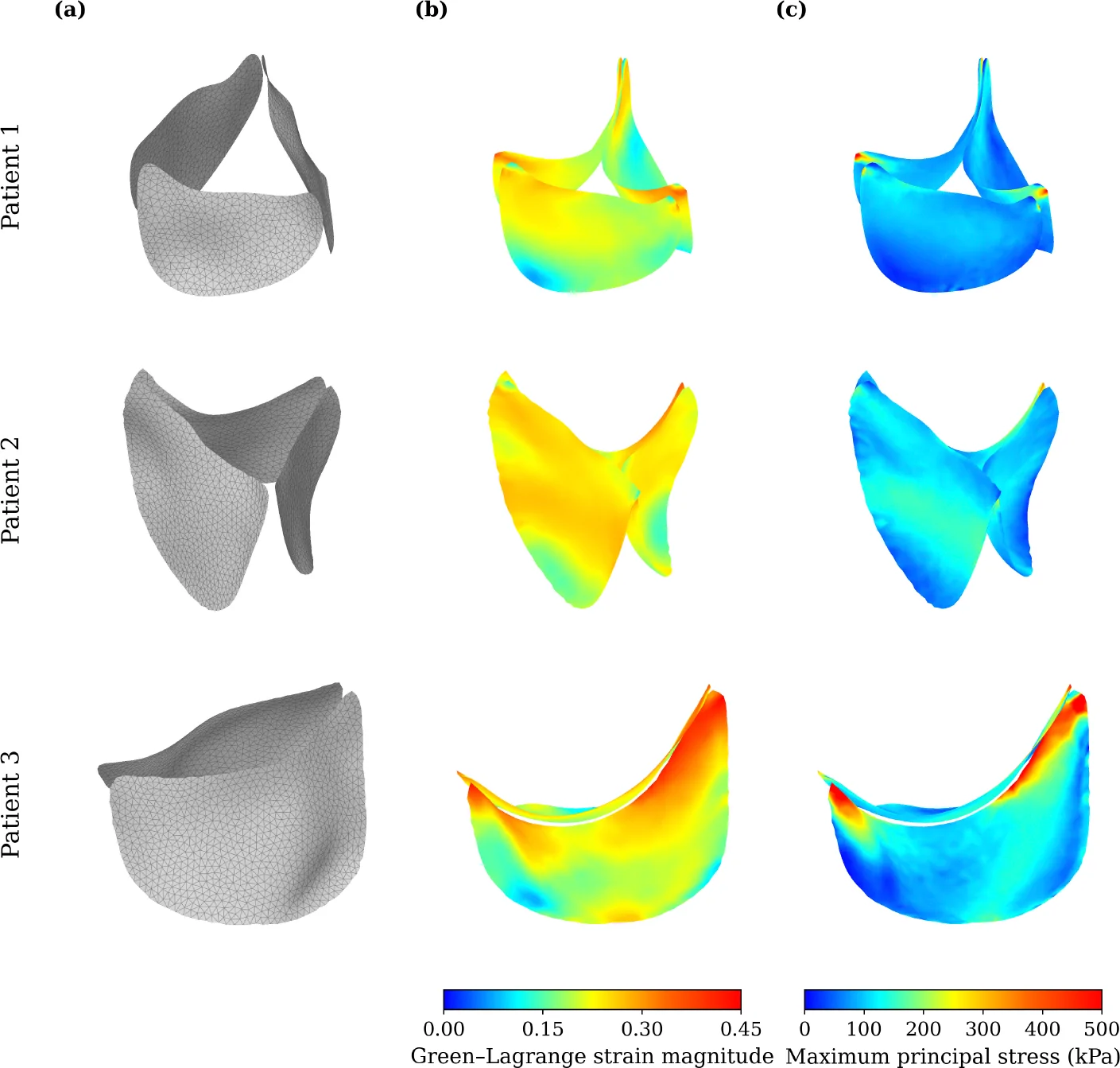
Patient-specific aortic-valve simulations. Rows show patient 1 (tricuspid), patient 2 (tricuspid), and patient 3 (bicuspid). Columns show (a) the open reference configuration, (b) Green–Lagrange strain magnitude and (c) estimated maximum principal Cauchy stress on the closed configurations at p=10kPa.

**Figure 8:**
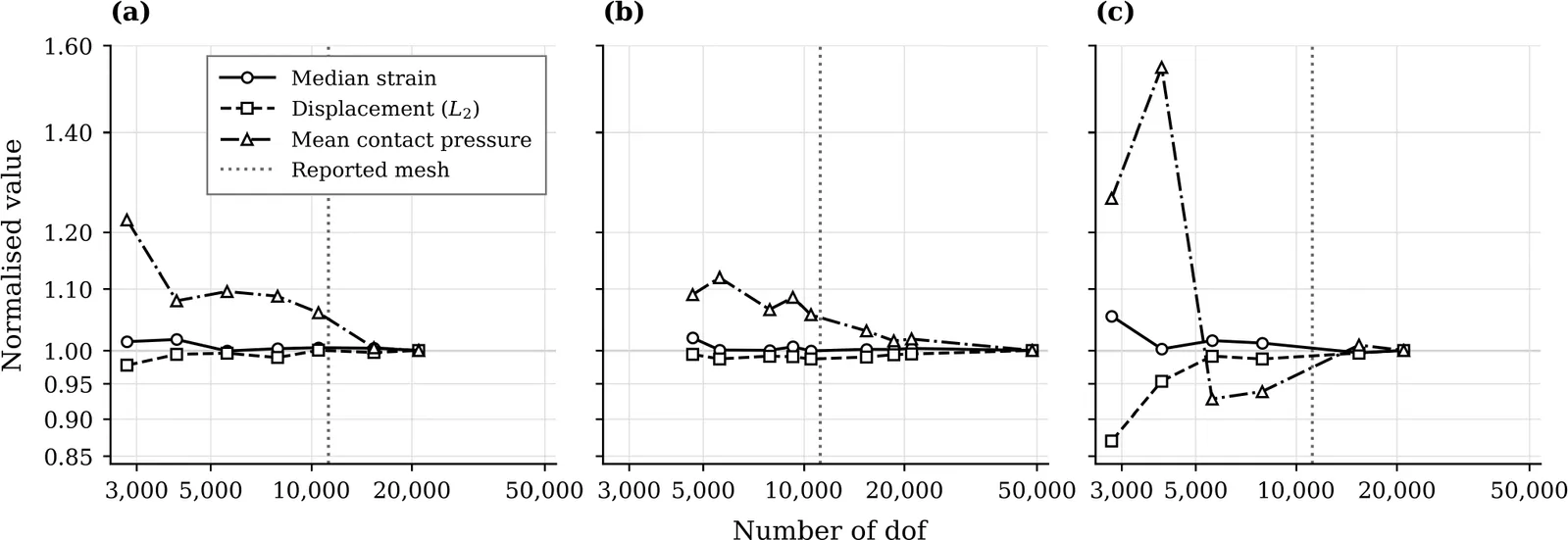
Mesh sensitivity for (a) patient 1, (b) patient 2, and (c) patient 3. Each quantity is normalized by its value on the patient’s finest accepted mesh. Displacement ($L_{2}$) denotes root-mean-square nodal displacement from the open reference to the closed configuration. Dotted lines mark the dof counts used for the reported simulations.

**Table 1:** Algorithm for the matrix-free Newton–Krylov solution with incremental loading and coupled full-load equilibrium refinement.

Step	Operation and objective

**Part 1: Advance to the target load.** For each load step, compute the pressure force and contact candidate lists from the previous accepted configuration and hold them fixed during the step. Internal equilibrium is approximated using limited Newton updates.	
**Repeat Steps 1–4:**	
1	**Update the thickness field.** At fixed U, apply global Newton updates to the thickness-field equations:
	$r_{w}=\frac{\partial {\tilde{\Pi }}_{h}}{\partial w}=0.$
2	**Update the EAS variables.** Apply local Newton updates to the internal equilibrium equation in each element:
	$r_{\alpha_{e}}=\frac{\partial {\tilde{\Pi }}_{h}}{\partial \alpha_{e}}=0,\;\forall e.$
3	**Compute the primary correction.** With the internal fields and pressure force fixed, use preconditioned CG and backtracking:
	$(K+\mu I)\Delta U=-r_{U},\;U\leftarrow U+s\Delta U.$
	Increase μ and retry if backtracking fails.
4	**Check the load step.** Refresh the internal fields at the updated U and re-evaluate the equilibrium residual.
	$\text{Until}\;\frac{\left\|r_{U}\right\|}{max(\left\|{\bar{F}}_{p}\right\|,F_{0})}<5\times 10^{-2}.$
	Accept the step and advance the load. If the iteration limit is reached, reduce the load increment and retry.

**Part 2: Converge the full-load equilibrium.** At the target pressure, refine the coupled fields and update the geometry-dependent pressure force.	
**Repeat Steps 5–6:**	
5	**Refine the coupled equilibrium.** Update the internal fields, eliminate the local EAS coupling, and use the reduced tangent:
	${\hat{K}}_{red}=K_{UU}^{\left(\alpha \right)}-K_{Uw}^{\left(\alpha \right)}{\left(K_{ww}^{\left(\alpha \right)}+\beta I\right)}^{-1}K_{wU}^{\left(\alpha \right)}.$
	Include the thickness-field residual in the right-hand side of the correction equations, apply the Levenberg shift μI, and update U and w together using backtracking.
6	**Update pressure forces and check convergence.** Recalculate $F_{p}=F_{p}(U)$ and re-evaluate the original equilibrium residual:
	$\text{Until}\;\frac{\left\|r_{U}\right\|}{max\left(\left\|F_{p}\right\|,F_{0}\right)}\leq 10^{-3}\text{,}$
	and the pressure-force and internal-variable convergence criteria are satisfied at the same updated state.

**Table 2:** Geometry and material parameters for the thick-walled cylinder verification.

Model	Parameters

Geometry and kinematics	*A* = 5 mm, *B* = 10 mm, *λ_z_* = 1
neo-Hookean	*μ* = 50 kPa, *λ* = 5 × 10^4^ kPa
Lee–Sacks	*c*_0_ = 50 kPa, *c*_1_ = 25 kPa, *c*_2_ = 5, *K* = 5 × 10^4^ kPa
HGO	*μ* = 50 kPa, *λ* = 5 × 10^4^ kPa, *k*_1_ = 75 kPa, *k*_2_ =4, *β* = 45°

**Table 3:** Patient-specific aortic-valve closure at 10 kPa. Peak RSS is the maximum host-process resident memory over accepted timed runs. CPU and GPU runs were performed sequentially on one node using 12 physical Intel Xeon Gold 5317 cores and one NVIDIA A100-SXM4-40GB.

	patient 1 (tricuspid)	patient 2 (tricuspid)	patient 3 (bicuspid)

number of leaflets	3	3	2
global dof	11 256	11 207	11 200
$\left(c_{0},c_{1},K\right)$ (kPa)		(67, 13, 5000)	
$c_{2}$ (dimensionless)		38	

final relative residual	8.31 × 10^−5^	6.95 × 10^−5^	6.19 × 10^−5^

median ‖E‖	0.19	0.23	0.21
maximum ‖E‖	0.37	0.36	0.45
median $\sigma_{1}$ (kPa)	81.00	101.20	108.80
maximum $\sigma_{1}$ (kPa)	222.90	191.40	494.50
maximum displacement (mm)	8.44	9.53	14.74

CPU wall time, median (min)	87.88	77.75	165.65
GPU wall time, median (min)	15.17	14.77	25.40
CPU/GPU speed-up	5.79	5.27	6.52
peak host RSS, CPU/GPU (GiB)	3.35/4.37	3.26/4.22	3.41/4.12

**Table 4:** Functionality demonstrated in selected biomechanical studies. Entries describe the reported applications rather than all capabilities of the underlying software. “Not demonstrated” refers to the reported examples; “not reported” indicates unspecified GPU use.

Study / application	Computational approach	Elements	Anisotropic hyperelasticity	Contact scope	Derivative calculations	GPU use

Finsberg et al. [51] Ventricles	FEniCS / dolfin-adjoint	Tetrahedral solids	Yes	Not demonstrated	Adjoint equilibrium gradients for parameter estimation	CPU
Sacks et al. [52] Myocardium; aortic leaflet	Energy-trained NNFE; JAX for leaflet	3D solids; NURBS leaflet	Yes	Not demonstrated	Neural-network training AD	Not reported
Liang et al. [15] Aorta	PyTorch-FEA / PyTorch	Hexahedral solids	Yes	Not demonstrated	Stress, stiffness, and inverse-loss AD	Yes (hybrid)
Meyer et al. [11] Trileaflet valve	Energy-trained NNFE / JAX	3D NURBS solids	Yes	Large-deformation multi-body contact; self-contact not demonstrated	Neural-network training AD	GPU-based; hardware unspecified
Present study Aortic valve	Matrix-free / JAX; tatva FE foundation	Extensible-director shells	Cylinder benchmark; isotropic valves	Large-deformation leaflet contact; self-contact in ring benchmark	Residual and tangent AD; contact-free plate equilibrium sensitivities; contact-energy derivatives	Yes (hybrid)

## Data Availability

Data supporting this study are available on reasonable request to the corresponding authors. Clinical imaging data are not publicly shared to protect patient confidentiality.

## Code availability

The code used in this study will be made available upon acceptance of the manuscript.
